# Changes in body weight and the risk of breast cancer in *BRCA1 *and *BRCA2 *mutation carriers

**DOI:** 10.1186/bcr1293

**Published:** 2005-08-19

**Authors:** Joanne Kotsopoulos, Olufunmilayo I Olopado, Parviz Ghadirian, Jan Lubinski, Henry T Lynch, Claudine Isaacs, Barbara Weber, Charmaine Kim-Sing, Peter Ainsworth, William D Foulkes, Andrea Eisen, Ping Sun, Steven A Narod

**Affiliations:** 1 Centre for Research in Women's Health, Women's College Hospital, University of Toronto, Toronto, ON, Canada; 2 Department of Nutritional Sciences, University of Toronto, ON, Canada; 3 Center for Clinical Cancer Genetics, University of Chicago, Chicago, IL, USA; 4 Epidemiology Research Unit, Research Centre, Centre Hospitalier de l'Universitaire Montréal, CHUM Hôtel Dieu and Département de Nutrition, Faculté du Médecine, Quebec, QC, Canada; 5 Hereditary Cancer Center, Pomeranian Medical University, Szczecin, Poland; 6 Department of Preventive Medicine and Public Health, Creighton University School of Medicine, Omaha, NE, USA; 7 Lombardi Cancer Center, Georgetown University Medical Center, Washington, DC, USA; 8 Abramson Family Cancer Research Institute, University of Pennsylvania, Philadelphia, PA, USA; 9 British Columbia Cancer Agency, Vancouver, BC, Canada; 10 London Regional Cancer Center, London, ON, Canada; 11 Departments of Medicine, Human Genetics, and Oncology, McGill University, Montréal, QC, Canada; 12 Sunnybrook and Women's College Health Sciences, Toronto, ON, Canada

## Abstract

**Background:**

Several anthropometric measures have been found to be associated with the risk of breast cancer. Current weight, body mass index, and adult weight gain appear to be predictors of postmenopausal breast cancer. These factors have been associated with a reduced risk of premenopausal breast cancer. We asked whether there is an association between changes in body weight and the risk of breast cancer in women who carry a mutation in either breast cancer susceptibility gene, *BRCA1 *or *BRCA2*.

**Methods:**

A matched case–control study was conducted in 1,073 pairs of women carrying a deleterious mutation in either *BRCA1 *(*n *= 797 pairs) or *BRCA2 *(*n *= 276 pairs). Women diagnosed with breast cancer were matched to control subjects by year of birth, mutation, country of residence, and history of ovarian cancer. Information about weight was derived from a questionnaire routinely administered to women who were carriers of a mutation in either gene. Conditional logistic regression was used to estimate the association between weight gain or loss and the risk of breast cancer, stratified by age at diagnosis or menopausal status.

**Results:**

A loss of at least 10 pounds in the period from age 18 to 30 years was associated with a decreased risk of breast cancer between age 30 and 49 (odds ratio (OR) = 0.47; 95% confidence interval (CI) 0.28–0.79); weight gain during the same interval did not influence the overall risk. Among the subgroup of *BRCA1 *mutation carriers who had at least two children, weight gain of more than 10 pounds between age 18 and 30 was associated with an increased risk of breast cancer diagnosed between age 30 and 40 (OR = 1.44, 95% CI 1.01–2.04). Change in body weight later in life (at age 30 to 40) did not influence the risk of either premenopausal or postmenopausal breast cancer.

**Conclusion:**

The results from this study suggest that weight loss in early adult life (age 18 to 30) protects against early-onset *BRCA-*associated breast cancers. Weight gain should also be avoided, particularly among *BRCA1 *mutation carriers who elect to have at least two pregnancies.

## Introduction

The inheritance of a deleterious mutation in either of the two breast cancer susceptibility genes, *BRCA1 *or *BRCA2*, has been associated with a lifetime risk of breast cancer of 45% to 87% [1,2]. Reports of increasing penetrance among women born in recent cohorts in comparison with those born in earlier years has prompted the search for factors that may influence the risk of cancer in genetically susceptible women [2-5]. To date, both genetic and non-genetic factors have been suggested to influence breast cancer risk in *BRCA1 *and *BRCA2 *mutation carriers, and many implicate estrogen-induced stimulation as a probable contributor (reviewed in [6]). Genetic risk factors include both the type and position of the mutation [7-9], as well as the presence of specific alleles of modifying genes [10-13]. Non-genetic or environmental factors include hormonal factors, in particular those related to estrogen exposure (reviewed in [6]). Reproductive factors that modify risk in *BRCA* carriers include breastfeeding, parity, and oral contraceptive use (reviewed in [14]).

The worldwide prevalence of obesity is rising [15]. Evidence from animal studies suggests that positive energy balance has a growth-promoting effect on tumours [16]. Numerous epidemiological studies have evaluated the role of various anthropometric risk factors in the etiology of breast cancer (reviewed in [17]). Collectively, the evidence suggests that the effects of body mass index (BMI) and of adult weight gain on the risk of breast cancer are dependent on menopausal status at diagnosis. There appears to be an inverse relation between both BMI and weight and the risk of premenopausal breast cancer; whereas there is a positive association between body weight, BMI, and adult weight gain on the risk of breast cancer after the menopause (reviewed in [17-20]). Birthweight and adult height have been associated with an increased risk of breast cancer in both menopausal strata (reviewed in [17-20]). Weight change that occurs at the time a woman is undergoing hormonal changes (i.e. puberty, pregnancy, menopause) has also been suggested to have an effect on risk [21,22]. Although various biological mechanisms by which weight may influence breast cancer risk have been proposed (reviewed in [17]), of particular relevance is an increase in circulating endogenous sex hormones, particularly estrogen [23]. Epidemiologic observations and laboratory studies suggest that sex hormones play an important role in *BRCA*-carcinogenesis and the current chemopreventive options available for *BRCA *carriers are based on the interruption of the estrogen-signalling pathway (reviewed in [6,24]).

Studies are needed to determine if the known anthropometric risk factors for sporadic breast cancer may also influence the penetrance of breast cancer in *BRCA *carriers. We performed a matched case–control study to investigate whether or not there is an association between changes in body weight and the risk of breast cancer in women with a deleterious *BRCA1 *or *BRCA2 *mutation. The identification of non-genetic modifiers of risk may be useful for preventing hereditary breast cancer.

## Materials and methods

### Study population and design

Eligible study subjects included women who were alive and known to be carriers of deleterious mutations of the *BRCA1 *or *BRCA2 *gene. These women were identified from 41 participating centers in five countries and were participants in previous and ongoing clinical research protocols at the host institutions. All study subjects received counselling and gave their written informed consent for genetic testing.

The study was approved by the institutional review boards of the host institutions. In most cases, testing was initially offered to women who had been affected with breast or ovarian cancer. When a *BRCA1 *or *BRCA2 *mutation was identified in a proband or her relative, genetic testing was offered to other at-risk women in the family. Mutation detection was performed using a range of techniques, but all nucleotide sequences were confirmed by direct sequencing of DNA. A woman was eligible for the current study when the molecular analysis established that she was a carrier of a pathogenic mutation. Most (>95%) of the mutations identified in the study subjects were nonsense mutations, deletions, insertions, or small frameshifts.

There was information on cancer history and mutation carrier status for a total of 3,291 women who carried *BRCA1 *or *BRCA2 *mutations and who provided information on weight at ages 18, 30, and 40. Potential case subjects were selected from among the study subjects with a diagnosis of invasive breast cancer. Case subjects were excluded if they had been diagnosed with ovarian cancer (29 women) or any other form of cancer (28 women) before being diagnosed with breast cancer, or if information about their menopausal status was missing (31 women). Control subjects were women who had never had breast cancer and who were carriers of a mutation in the *BRCA1 *or *BRCA2 *gene. A subject was not eligible to be a control for a given case subject if she had had a protective bilateral mastectomy before the date of diagnosis in the case (88 women). After exclusions, there was a total of 3,115 eligible women, including 1,471 women with breast cancer (potential case subjects) and 1,644 women without breast cancer (potential controls).

A single control subject was selected for each case subject, matched according to mutation in the same gene (*BRCA1 *or *BRCA2*), year of birth (within 1 year), and the country of residence. A diagnosis of ovarian or other form of cancer in the control had to be after the year of diagnosis of breast cancer of the matched case subject for her to be eligible. In addition, the date of interview of the control was after the date of breast cancer diagnosis of the matched case. A total of 1,073 matched case–control pairs was generated for the analysis, including 797 pairs with *BRCA1 *mutations and 276 pairs with *BRCA2 *mutations. The 2,146 study subjects included in the analysis were identified from 1,534 distinct families (1.4 subjects per family). In the instance of 1,179 subjects, the subject was the only member of the family to be included. These were prevalent cases and had breast cancer before they knew their mutation status. On average, 8.8 years had passed between the subject's age at diagnosis (mean 39.8 years) and age at interview (mean 48.6 years).

### Data collection

Case and control subjects completed a questionnaire that asked for relevant information regarding family history, reproductive and medical histories, and selected lifestyle factors including smoking history and use of oral contraceptives. Questionnaires were administered by each of the individual centers at the time of a clinic appointment or at their home at a later date. Interviews occurred between 1988 and 2004 for the case subjects and between 1994 and 2004 for the control subjects. Additional variables of interest included information on demography, ethnicity, and parity. Women were classified as postmenopausal if they reported natural menopause and had stopped menstruating, or if they had had a hysterectomy and bilateral oophorectomy before the diagnosis of breast cancer. Specifically for this study, the questionnaire asked for information on height (in feet and inches) and weight (in pounds). The participants were requested to think back to when they were 18 years old (about the time they graduated from high school) and to recall their weight then and subsequently at ages 30 and 40. Women were asked to report their weight at birth, their current weight, and their height, as well as the most they had ever weighed (excluding pregnancy). Only case and control data before the time of the diagnosis of breast cancer in the matched case were considered.

### Anthropometric measures

We converted the reported weights from pounds to kilograms and the heights from inches to meters for BMI calculations. Variables that were created in this study included BMI (weight (kg)/height(m^2^)) at ages 18, 30, and 40 years, and weight change between age 18 and 30 and between 30 and 40 (calculated as the difference between the weights at the age periods being compared).

### Statistical analyses

A matched case – control analysis was performed to examine the association between weight and changes in body weight, and the risk of breast cancer. Because menopausal status has been shown to modify the association between anthropometric factors and the risk of breast cancer, our analyses were stratified according to menopausal status at the time the subject received a diagnosis of breast cancer diagnosis. Birthweight, height, weight, weight gain, and BMI were compared between the case subjects and control subjects within each stratum, using a paired *t*-test. This test statistic was also used for all other continuous variables that were examined. The χ^2 ^test was used to test for differences in categorical variables. The univariate odds ratios (ORs), 95% confidence intervals (CIs), and tests for linear trend were estimated by use of conditional logistic regression. A multivariate analysis was also carried out to control for the potential confounding effects of oral contraceptive use, smoking, oophorectomy, and parity. Smoking use was coded as 'ever' or 'never' smoker; oral contraceptive use was coded as 'ever' or 'never' user; oophorectomy was coded as yes or no; and parity was coded as zero, one, or two or more births. Weight change was categorized into quartiles according to the distribution of the variables among the controls.

The reference group were those women whose weight remained stable (weight gain or loss of not more than 10 pounds from baseline). The weight-loss group included women who lost at least 10 pounds. We examined the effect of weight change between ages 18 and 30 and between ages 30 and 40 among subgroups defined according to the subject's age at diagnosis of the case. This effect was further evaluated according to mutation and menopausal status. There were 26 menopausal case subjects who reported having had a hysterectomy before their breast cancer had been diagnosed but who still had intact ovaries. These 26 pairs were excluded from the subanalyses stratified by menopausal status. Odds ratios were generated for these subgroups with the matched-pair subsets. All statistical tests were two-sided. A *P *value of <0.05 was taken to be significant. All analyses were performed using the SAS statistical package, version 8.1 (SAS Institute, Cary, NC, USA).

## Results

### Study subjects

Case and control subjects were similar with regard to year of birth, year of interview, current age, mutation status, smoking history, and country of residence (Table 1). Oral contraceptives had been used by more of the case than control subjects (*P *= 0.04), and parity was also slightly higher in the case than control subjects (*P *= 0.06).

### Comparison of anthropometric measures in *BRCA1 *or *BRCA2 *mutation carriers

Table 2 compares the mean values for various anthropometric measures for the cases and controls as a whole, and stratified by the menopausal status of the case subject when the breast cancer was diagnosed. Among all the study participants, case subjects weighed less at age 18 than the control subjects. Among postmenopausal women, case subjects had a lower BMI at age 18 than controls. There were no other statistically significant differences between the case and control subjects with respect to weight at birth, current height, weight, BMI, or weight gain at various ages (Table 2).

**Figure 1:**
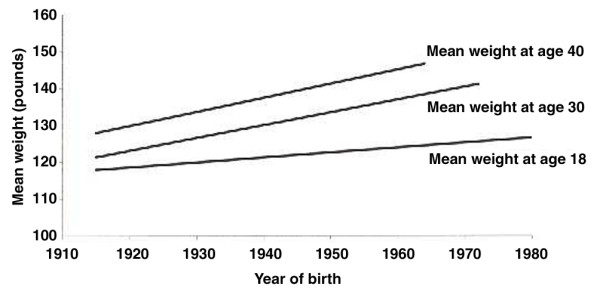
Weight at various ages among *BRCA *mutation carriers according to year of birth.

The extent of weight gain experienced by our study subjects varied according to their year of birth (Fig. 1). Those born in earlier years experienced on average less weight gain between age 18 and 30 and between 18 and 40 than women born in later years. The increase in weight by calendar year is most apparent at the ages of 30 and 40. There was also a significant difference between the mean weights at ages 18, 30, and 40 among women residing in Canada, Poland, or the USA (*P *= 0.0001, 0.02, and 0.02, respectively).

### Changes in body weight between age 18 and 30 and risk of breast cancer in *BRCA *mutation carriers

To further examine the relationship between adult weight change and the risk of breast cancer, we performed univariate conditional logistic regression. The adjusted ORs were similar to the unadjusted values; therefore, only univariate results are reported here. As Table 3 shows, weight loss of at least 10 pounds between age 18 and 30 was associated with a significant reduction in breast cancer risk thereafter (OR = 0.66). Weight gain during this period was not associated with increased risk. However, stratification of the study subjects according to their age at breast cancer diagnosis indicated that changes in body weight appeared to have different effects in carriers according to whether the breast cancer was diagnosed before or after age 40. Weight loss of at least 10 pounds was associated with a significant reduction in the risk of breast cancer diagnosed between age 30 and 40 (OR = 0.47) (Table 3) but was not associated with the risk of breast cancer diagnosed after age 40.

Subgroup analyses according to *BRCA *mutation status showed that among women with a *BRCA1 *mutation, weight loss of at least 10 pounds was associated with a 65% reduction in cancer risk compared with women in the reference group (OR = 0.35) (Table 4). A modest protective effect of this degree of weight loss was also seen among *BRCA2 *mutation carriers, although this association did not reach statistical significance (OR = 0.88).

The mean baseline weight (weight at age 18) of the *BRCA1 *mutation carriers who lost more than 10 pounds was 142.5 pounds (range 115 to 230 pounds). These women experienced a mean weight loss of 18.6 pounds (range 10 to 86 pounds) between age 18 and 30. Forty percent of these women had a mean baseline weight greater than 150 pounds and 35% had a BMI greater than 25.

### Changes in body weight between age 18 and 30, parity, and risk of breast cancer in *BRCA *mutation carriers

Because parity has been shown to modify the risk of breast cancer in carriers [25], we next examined the risk of breast cancer associated with weight gain but taking into account the possible modifying effect of parity (Table 5). Compared with those who experienced minimal changes in body weight (± 10 pounds), weight gain of greater than 10 pounds among women who had at least two full-term pregnancies was significantly associated with an increase in the risk of breast cancer (OR = 1.44). To discern whether increased parity *per se* was associated with weight gain, we compared mean weight gain among the carriers, according to parity. The mean weight gain across the groups was similar (data not shown). Therefore, the increased risk of breast cancer associated with parity and any weight gain is not attributable to greater weight gain among those who had higher parity. A modifying effect of parity and weight gain was not seen among women with a *BRCA2 *mutation (Table 5).

## Discussion

We conducted our study to examine whether change in body weight modifies the risk of breast cancer among women who carry a deleterious *BRCA1 *or *BRCA2 *mutation. We found that *BRCA *mutation carriers who lost at least 10 pounds between age 18 and 30 had a 34% reduction in the risk of breast cancer. However, on stratification of the sample by age of breast cancer diagnosis, this protective effect was only observed among *BRCA *mutation carriers diagnosed between age 30 and 40 and not for those diagnosed after age 40. Although weight loss reduced the risk of breast cancer among carriers of either mutation, this association remained significant only for women with a *BRCA1 *mutation (OR = 0.35). A large proportion of the group who experienced weight loss had a baseline BMI of greater than 25, the BMI cut-point for the classification of overweight individuals [26]. This suggests that recommendations regarding weight loss should be targeted towards those women who are considered to be overweight at age 18.

The role of early adult weight gain and subsequent risk of breast cancer is not well defined. The majority of studies report either no association or a decrease in risk with weight gain for premenopausal women, and inconsistent results for postmenopausal women [19,21]. It has been suggested that adult weight gain may be a better measure of adiposity than BMI, because lean body mass decreases with age [27] and BMI does not distinguish between lean and fat mass; whereas changes in adult weight largely reflect changes in body fat [19,28]. Adult weight gain appears to be a consistent and independent predictor of postmenopausal breast cancer risk, particularly in women who never used hormone replacement therapy [21,29-31]. Studies of adult weight gain and the risk of premenopausal breast cancer have generally shown a reduction in risk, although two studies found no association [21,32]. In our selected study population as a whole, weight gain did not influence risk. Rather, we observed a decrease in the risk of breast cancer diagnosed between age 31 and 40 associated with weight loss in early adulthood (between age 18 and 30). Weight change that occurred between age 30 and 40 did not influence the subsequent risk of either premenopausal or postmenopausal breast cancer. Our findings suggest an important effect of weight loss in early years and the risk of early-onset breast cancer. This effect is of particular relevance to our study population, because a characteristic feature of *BRCA*-associated breast cancers is young age at diagnosis [33].

Our findings suggest that in *BRCA *carriers, changes in body weight throughout early adult life may have a more important influence on the risk of early-onset breast cancer than current weight or BMI [21]. The magnitude of the decreased risk associated with weight loss compared with those women whose weight remained stable was relatively large (OR = 0.47) (see Table 3). After stratification by mutation status, the protective effect of weight loss between age 18 and 30 was seen to be less strong among women with a *BRCA2 *mutation. These findings suggest that the timing of weight loss may play a more important role in *BRCA1*-associated than in *BRCA2*-associated carcinogenesis, though the lack of a significant finding for the latter group might also be attributable to a smaller sample size. The effect may be of greater importance for women belonging to more recent birth cohorts, since there appears to be a greater increase in average weight at ages 30 and 40 with each decade (see Fig. 1).

We also found that in the subgroup of *BRCA1 *mutation carriers who gained 10 pounds or more and who had at least two full-term pregnancies, there was a 44% increase in their risk of breast cancer. The modifying effects of both parity and weight gain were not observed for women with a *BRCA2 *mutation. The number of births did not influence the amount of weight gain experienced by either the case or the control subjects, providing confirmation that weight gain is not a surrogate for parity or vice versa. Although pregnancy itself offers long-term protection against postmenopausal breast cancer in the general population, significant weight gain during pregnancy has been associated with an increased risk of developing breast cancer after the menopause [34]. We have reported elsewhere that parity is a risk factor for breast cancer in *BRCA2 *carriers but not in *BRCA1 *carriers [25].

Ballard-Barbash proposed that weight change that occurs during periods of noticeable hormonal change (i.e. menarche, pregnancy, and menopause) may be attributed to host metabolic factors that may also influence breast cancer risk [21]. In addition, weight gain may result in differing biological effects depending on the body fat distribution [21]. Weight gain during pregnancy is characterized by an increase in central body fat deposition [35]. The physiological consequences of upper or central body fat localization include altered ovarian hormone and glucose metabolism, as well as insulin resistance and hyperinsulinemia, all of which may increase breast cancer risk [21,36]. This pattern of fat distribution has been suggested to pose a higher risk of breast cancer, independent of weight [22,37].

Only two studies have evaluated the association between anthropometric risk factors or physical activity and the risk of breast cancer in *BRCA1 *and/or *BRCA2 *carriers [4,38]. King and colleagues recently reported that a healthy weight defined at menarche and at age 21, as well as physical activity during adolescence, were associated with a significant delay in the age of onset of breast cancer in *BRCA1 *and *BRCA2 *carriers; however, such an effect could be attributable to either weight gain increasing the risk of early-onset breast cancer or to weight gain protecting against late-onset breast cancer [4]. An earlier study of 46 *BRCA1 *carriers found no significant effect of current BMI on the age at disease onset; however, the sample size was small [38].

The role of sex hormones in the etiology of breast cancer has been well established [23]. It is generally agreed that increasing levels of circulating estrogen are a determinant of obesity-associated breast cancer in postmenopausal women [39]. In contrast, most investigations of premenopausal women report an inverse association between weight (or BMI or weight gain) and the risk of breast cancer. The epidemiological evidence suggests a positive association between these anthropometric variables and the risk of postmenopausal breast cancer (reviewed in [17]). The primary hypothesis underlying this relation between menopausal status and the risk of breast cancer is believed to involve an alteration in the source and levels of endogenous sex hormones [19,40]. Before menopause, the ovaries are the primary site of endogenous hormone production. Since obesity has been shown to induce chronic anovulatory cycles and subsequently lower serum estrogen [41] and progesterone levels [42], a decrease in hormone exposure is believed to be the primary mechanism by which overweight women may be protected against premenopausal breast cancer [43]. Extraglandular aromatization of androstenedione to estrone occurs in the adipose tissue and is the primary source of estradiol in postmenopausal women [39]. This conversion of androgens and subsequent increase in estrogen levels has been shown to be directly proportional to the amount of adipose tissue [44] and the induction of aromatase activity which may possibly enhance estrogen production in adipose tissue [45]. In contrast, among *BRCA *carriers, weight gain did not affect the risk of breast cancer.

Other metabolic consequences of obesity, more specifically central adiposity, that have been suggested to be factors in the development of breast cancer include hyperinsulinemia and insulin resistance, as well as elevated levels of glucose and triglycerides [46-50]. Obesity has also been shown to increase testosterone [51,52] and leptin levels, [53,54] and to depress sex-hormone-binding globulin concentrations. This globulin is the predominant carrier of estradiol levels in both premenopausal and postmenopausal women and is the primary protein responsible for binding and inactivating estradiol [41,55,56]. Therefore, reducing the concentration of sex-hormone-binding globulin may lead to an increase in the amount of unbound, free estradiol.

High concentrations of circulating insulin-like growth factor1 (IGF-1) appears to be a risk factor for premenopausal breast cancer in the general population, yet no such relation has been observed for postmenopausal breast cancer [57]. Studies have shown that both insulin and IGF-1 exert a mitogenic effect by stimulating cell proliferation and inhibiting apoptosis of breast cancer cells [58,59]. More importantly, it has been suggested that IGF-1 may also work synergistically with other growth factors and hormones, including estrogen, to further promote cell proliferation [60]. Although both BMI and IGF-1 levels are suggested to influence breast cancer risk, studies have generally shown no association or an inverse association between BMI and circulating IGF-1 levels [60].

Both birthweight [61,62] and height [63] are positively associated with IGF-1 levels. The evidence, primarily from cohort studies, supports a positive association between birthweight and the risk of breast cancer (reviewed in [64]) suggesting that prenatal events may influence later risk. Adult height has also been shown to positively predict the risk of breast cancer in both pre- and postmenopausal women [18,32]. In our study, there was no significant difference in birthweight between the cases and controls and it seems unlikely that this variable influences risk in *BRCA *mutation carriers. Current height was not associated with the risk of breast cancer, and this observation is in agreement with a pooled analysis of 52 epidemiological studies whereas height did not modify risk in women who had one or more affected first-degree relatives in comparison with women who had no affected relatives [65].

A potential drawback of our study was the use of self-reported risk factor data, which may have introduced measurement error and led to a spurious result or attenuation of results. However, validation studies have shown that current and recalled self-reported weight and height measurements are highly correlated with measured data [66-72]. Self-reporting many years prior has still been shown to retain a high degree of validity [19]. Our data was collected on average 9 years after the breast cancer diagnosis of the case, and 30 years after age 18 (the first weight reported). There is a potential for recall bias but there is no evidence of this in Table 2. The mean weights at each reported age were similar and the differences were not significant. In fact, the reported weight at age 18 was less for cases than controls (we might expect recall bias to generate the opposite result). Also, the dissimilar results for *BRCA1 *and *BRCA2 *carriers argues against recall bias.

Despite the primary limitation of recall bias and other inherent limitations associated with the use of case–control studies, the primary strength of our study is the large sample of known *BRCA *mutation carriers. This study involved 1,073 matched pairs selected from a total of approximately 3,291 documented mutation carriers and is by far the largest study addressing the role of anthropometric measures on the risk of hereditary breast cancer. Our matching strategy and exclusion criteria resulted in case and control groups that were similar in most respects. We believe that our study participants are representative of women who have had *BRCA *mutations identified during the course of genetic counselling. Our study was based on known mutation carriers and included patients from numerous participating centers and of different ethnic backgrounds.

## Conclusion

Our findings suggest that weight loss in early adult life (and not weight *per se*) decreases the risk of *BRCA*-associated breast cancer diagnosed at an early age. More specifically, the period between age 18 and 30 years appears to be a critical one when weight gain should be avoided in mutation carriers. The effect may be greatest in *BRCA1 *carriers experiencing at least two full-term pregnancies, but further study is necessary to confirm this subgroup analysis. The maintenance of a healthy weight during early adult life represents a potentially modifiable risk factor in hereditary breast cancer syndromes.

## Abbreviations

*BRCA1 *= breast cancer susceptibility gene 1; *BRCA2 *= breast cancer susceptibility gene 2; CI = confidence interval; IGF-1 = insulin-like growth factor 1**; **OR = odds ratio.

## Competing interests

The authors declare that they have no competing interests.

## Authors' contributions

SAN conceived and designed the study. JK drafted the manuscript and helped with the analysis. PS performed the statistical analysis. OIO, PG, JL, HTL, CI, BW, CK-S, PA, WDF, and AI coordinated study activities for their centers and helped with the preparation of the manuscript. All authors read and approved the final version of the manuscript.

## Figures and Tables

**Table 1:** Comparison of subjects carrying *BRCA *mutations

Variable	Control subjects (*n *= 1,073)	Case subjects (*n *= 1,073)	*P*^a^
Age (years) at interview, no. (%)			
≤ 30	17 (1.6)	26 (2.4)	
31–40	219 (20.4)	242 (22.5)	
41–50	415 (38.7)	386 (36.0)	
51–60	277 (25.8)	264 (24.6)	
≥ 61	145 (13.5)	155 (14.5)	0.32
Age (years) at interview, mean (SD)	47.9 (10.6)	48.6 (10.6)	0.25
Date of birth, mean year	1951.8	1951.2	0.14
Year of interview, mean (range)	1999.7 (1995–2004)	1999.8 (1999–2004)	0.18
			
Age (years) at diagnosis of breast cancer, no. (%)			
≤ 30	NA	107 (10.0)	
31–40		478 (44.5)	
41–50		371 (34.5)	
≥ 51		117 (10.9)	
Age (years) at diagnosis of breast cancer, mean (SD)		39.8 (8.3)	
			
Mutation, %			
*BRCA1*	74.3	74.3	
*BRCA2*	25.7	25.7	
			
Parity			
No. (%) parous	844 (79.6)	879 (82.8)	0.06
Parity, mean (SD)	1.9 (1.4)	1.9 (1.3)	0.18
			
No. (%) who ever used oral contraceptives	662 (62.6)	710 (66.8)	0.04
No. (%) who ever smoked	465 (43.3)	471 (43.9)	0.79
			
Country of residence at time of testing, no. (%)			
Canada	420 (39.1)	420 (39.1)	
Israel	20 (1.9)	20 (1.9)	
UK	8 (0.8)	8 (0.8)	
Poland	189 (17.6)	189 (17.6)	
USA	436 (40.6)	436 (40.6)	

^a^All *P *values are univariate and were derived using Student's *t*-test for continuous variables and the χ^2 ^test for categorical variables. NA, not applicable; SD, standard deviation.

**Table 2:** Anthropometric variables in women with a deleterious *BRCA1 *or *BRCA2 *mutation^a^

	All			Premenopausal			Postmenopausal		
Variable	Cases * n *= 1073	Controls *n *= 1073	*P*	Cases *n *= 817	Controls *n *= 817	*P*	Cases *n *= 256	Controls *n *= 256	*P*
Weight (pounds)									
At birth	7.1 (1.3)	7.1 (1.3)	0.67	NA	NA	NA	NA	NA	NA
At age 18	120.6 (17.2)	122.3 (19.1)	0.03	121.5 (17.1)	122.9 (19.2)	0.11	117.8 (17.3)	120.4 (18.5)	0.10
At age 30^c^	130.9 (17.3)	131.2 (18.5)	0.75	131.9 (17.1)	132.2 (18.5)	0.87	127.9 (17.4)	128.6 (18.6)	0.69
At age 40^d^	137.7 (23.4)	137.7 (24.3)	0.99	139.7 (22.8)	138.7 (25.0)	0.63	135.3 (23.9)	136.5 (23.4)	0.59
Current height (inches)	64.2 (2.6)	64.3 (2.6)	0.44	64.3 (2.6)	64.5 ± 2.6	0.50	63.9 ± 2.7	63.7 ± 2.7	0.10
Body mass index^b^									
At age 18 (kg/m^2^)	20.57 (2.8)	20.77 (3.0)	0.10	20.65 (2.8)	20.74 (3.0)	0.52	20.31 (2.7)	20.88 (2.9)	0.03
At age 30 (kg/m^2^)^c^	22.35 (3.4)	22.32 (3.7)	0.88	22.44 (3.4)	22.33 (3.9)	0.56	22.08 (3.4)	22.31 (3.1)	0.43
At age 40 (kg/m^2^)^d^	23.77 (3.9)	23.73 (3.9)	0.88	24.06 (3.8)	23.67 (4.1)	0.26	23.42 (4.0)	23.80 (3.7)	0.29
Weight gain (pounds)									
From age 18 to 30^c^	10.5 (15.2)	9.4 (17.0)	0.14	10.6 (15.1)	9.8 (18.2)	0.35	10.1 (15.7)	8.2 (13.0)	0.23
From age 30 to 40^d^	9.4 (12.5)	9.2 (13.3)	0.82	10.6 (12.5)	9.1 (15.3)	0.23	7.9 (12.3)	8.2 (10.5)	0.23
From age 18 to 40^d^	18.6 (19.5)	18.1 (20.4)	0.71	19.9 (20.2)	19.0 (22.1)	0.61	17.0 (18.6)	17.1 (18.2)	0.98

The subjects were women who did (case subjects) or did not (control subjects) have a diagnosis of invasive breast cancer. ^a^*P *values are univariate and were derived using Student's *t*-test and include both *BRCA1 *and *BRCA2 *mutation carriers. Other values are means (standard deviations). ^b^Excludes subjects with missing data on current height. ^c^Calculated for pairs in which case subjects were diagnosed at age >30 years. ^d^Calculated for pairs in which case subjects were diagnosed at age >40 years. NA, not applicable.

**Table 3:** Weight change and subsequent cancer risk: subjects stratified by their age at diagnosis of cancer

Weight change between age 18 and 30 years	Cases (number)	Controls (number)	OR^a ^(95% CI)	*P*	*P *for trend
In all subjects^b^	966	966			
Loss of at least 10 pounds	53	81	0.66 (0.46–0.93)	0.03	
Loss of <10 to gain of ≤ 10 pounds	536	542	1 (referent)		
Gain of 10 to ≤ 20 pounds	227	190	1.19 (0.96–1.49)	0.12	
Gain of > 20 pounds	150	135	1.00 (0.77–1.30)	0.99	0.46
According to case subjects' age at diagnosis					
>30 to ≤ 40 years^c^	478	478			
Loss of at least 10 pounds	23	49	0.47 (0.28–0.79)	0.005	
Loss of <10 to gain of ≤ 10 pounds^d^	255	254	1 (referent)		
Gain of 10 to ≤ 20 pounds	112	89	1.25 (0.91–1.71)	0.17	
Gain of >20 pounds	88	86	1.03 (0.72–1.47)	0.88	0.48
>40 years^d^	488	488			
Loss of at least 10 pounds	30	32	0.97 (0.52–1.65)	0.91	
Loss of <10 to gain of ≤ 10 pounds^d^	281	288	1 (referent)		
Gain of 10 to ≤ 20 pounds	115	101	1.16 (0.85–1.59)	0.36	
Gain of >20 pounds	62	67	0.95 (0.64–1.43)	0.82	0.75

Subjects were women with a deleterious mutation in *BRCA1 *or *BRCA2 *who did (case subjects) or did not (control subjects) receive a diagnosis of cancer. ^a^All odds ratios (ORs) were derived using univariate conditional logistic regression. ^b^Excludes case subjects diagnosed at age ≤ 30 years. ^c^Excludes case subjects diagnosed at age ≤ 30 and >40 years. ^d^Excludes case subjects diagnosed at ≤ 40 years. CI, confidence interval.

**Table 4:** Weight change and subsequent cancer risk: subjects stratified by their *BRCA *mutation

Weight change between age 18 and 30 years	Cases (n)	Controls (n)	OR^a ^(95% CI)	*P*	*P *for trend
In *BRCA1 *mutation carriers	370	370			
Loss of at least 10 pounds	13	38	0.35 (0.18–0.67)	0.002	
Loss of <10 to gain of ≤ 10 pounds^d^	188	189	1 (referent)		
Gain of 10 to ≤ 20 pounds	93	72	1.29 (0.91–1.83)	0.15	
Gain of >20 pounds	76	71	1.09 (0.73–1.62)	0.67	0.34
In *BRCA2 *mutation carriers	108	108			
Loss of at least 10 pounds	10	11	0.88 (0.35–2.23)	0.78	
Loss of <10 to gain of ≤ 10 pounds^d^	67	65	1 (referent)		
Gain of 10 to ≤ 20 pounds	19	17	1.08 (0.50–2.35)	0.84	
Gain of >20 pounds	12	15	0.77 (0.33–1.81)	0.55	0.70

Subjects were women with a deleterious mutation in *BRCA1 *or *BRCA2 *who did (case subjects) or did not (matched control subjects) receive a diagnosis of breast cancer at age 30 to 39 years. ^a^All odds ratios (ORs) were derived using univariate conditional logistic regression. CI, confidence interval.

**Table 5:** Weight change and subsequent cancer risk: subjects^a ^stratified by *BRCA *mutation and parity

Weight change between age 18 and 30 years	Cases (number)	Controls (number)	OR^c ^(95% CI)	*P*	*P *for trend^d^
In *BRCA1 *mutation carriers					
Loss of <10 to gain of ≤ 10 pounds^b^	188	189	1 (referent)		
Gain of >10 pounds					
Parity = 0	24	28	0.88 (0.50–1.55)	0.66	
Parity = 1	26	27	0.94 (0.52–1.72)	0.85	
Parity ≥ 2	117	84	1.44 (1.01–2.04)	0.04	0.16
Parity unknown	2	4			
In *BRCA2 *mutation carriers					
Loss of <10 to gain of ≤ 10 pounds^b^	67	65	1 (referent)		
Gain of >10 pounds					
Parity = 0	6	4	1.44 (0.40–5.13)	0.58	
Parity = 1	7	6	1.10 (0.33–3.72)	0.87	
Parity ≥ 2	18	22	0.73 (0.33–1.64)	0.45	0.31
Parity unknown	0	0			

^a^Whose cancer was diagnosed when they were 30 to 39 years old. ^b^A negative number indicates weight loss. ^c^All ORs were derived using univariate conditional logistic regression. ^d^Excludes subjects in the weight-gain ≤ -10 group.

## References

[B1] Ford D, Easton DF, Stratton M, Narod S, Goldar D, Devilee P, Bishop DT, Weber B, Lenoir G, Chang-Claude J (1998). Genetic heterogeneity and penetrance analysis of the BRCA1 and BRCA2 genes in breast cancer families. The Breast Cancer Linkage Consortium. Am J Hum Genet.

[B2] Antoniou A, Pharoah PD, Narod S, Risch HA, Eyfjord JE, Hopper JL, Loman N, Olsson H, Johansson O, Borg A (2003). Average risks of breast and ovarian cancer associated with BRCA1 or BRCA2 mutations detected in case Series unselected for family history: a combined analysis of 22 studies. Am J Hum Genet.

[B3] Narod S, Lynch H, Conway T, Watson P, Feunteun J, Lenoir G (1993). Increasing incidence of breast cancer in family with BRCA1 mutation. Lancet.

[B4] King MC, Marks JH, Mandell JB (2003). Breast and ovarian cancer risks due to inherited mutations in BRCA1 and BRCA2. Science.

[B5] Foulkes WD, Brunet JS, Wong N, Goffin J, Chappuis PO (2002). Change in the penetrance of founder BRCA1/2 mutations? A retrospective cohort study. J Med Genet.

[B6] Narod SA (2002). Modifiers of risk of hereditary breast and ovarian cancer. Nat Rev Cancer.

[B7] Gayther SA, Warren W, Mazoyer S, Russell PA, Harrington PA, Chiano M, Seal S, Hamoudi R, van Rensburg EJ (1995). Germline mutations of the BRCA1 gene in breast and ovarian cancer families provide evidence for a genotype-phenotype correlation. Nat Genet.

[B8] Gayther SA, Mangion J, Russell P, Seal S, Barfoot R, Ponder BA, Stratton MR, Easton D (1997). Variation of risks of breast and ovarian cancer associated with different germline mutations of the BRCA2 gene. Nat Genet.

[B9] Thompson D, Easton D (2001). Variation in cancer risks, by mutation position, in BRCA2 mutation carriers. Am J Hum Genet.

[B10] Rebbeck TR, Kantoff PW, Krithivas K, Neuhausen S, Blackwood MA, Godwin AK, Daly MB, Naraod SA, Garber JE, Lynch HT (1999). Modification of BRCA1-associated breast cancer risk by the polymorphic androgen-receptor CAG repeat. Am J Hum Genet.

[B11] Rebbeck TR, Wang Y, Kantoff PW, Krithivas K, Neuhausen SL, Godwin AK, Daly MB, Narod SA, Brunet JS, Vesprini D (2001). Modification of BRCA1- and BRCA2-associated breast cancer risk by AIB1 genotype and reproductive history. Cancer Res.

[B12] Wang WW, Spurdle AB, Kolachana P, Bove B, Moden B, Ebbers SM, Suthers G, Tucker MA, Kaufman DJ, Doody MM (2001). A single nucleotide polymorphism in the 5' untranslated region of RAD51 and risk of cancer among BRCA1/2 mutation carriers. Cancer Epidemiol Biomarkers Prev.

[B13] Levy-Lahad E, Lahad A, Eisenberg S, Dagan E, Paperna T, Kasinetz L, Catane R, Kaufman B, Beller U, Renbaum B (2001). A single nucleotide polymorphism in the RAD51 gene modifies cancer risk in BRCA2 but not BRCA1 carriers. Proc Natl Acad Sci USA.

[B14] Narod SA, Offit K (2005). Prevention and management of hereditary breast cancer. J Clin Oncol.

[B15] International Obesity Task Force. http://www.iotf.org/.

[B16] Tannenbaum A (1996). The dependence of tumor formation on the composition of the calorie-restricted diet as well as on the degree of restriction. 1945. Nutrition.

[B17] Friedenreich CM (2001). Review of anthropometric factors and breast cancer risk. Eur J Cancer Prev.

[B18] van den Brandt PA, Spiegelman D, Yaun SS, Adami HO, Beeson L, Folsom AR, Fraser G, Goldbohm RA, Graham S, Kushi L (2000). Pooled analysis of prospective cohort studies on height, weight, and breast cancer risk. Am J Epidemiol.

[B19] Vainio H, Bianchini F, (Eds) (2002). Weight Control and Physical Activity.

[B20] Berglund G, Riboli EL (2002). Anthropometry, physical activity and cancer of the breast and colon. Nutrition and Lifestyle: Opportunities for Cancer Prevention.

[B21] Ballard-Barbash R (1994). Anthropometry and breast cancer. Body size – a moving target. Cancer.

[B22] Kumar NB, Lyman GH, Allen K, Cox CE, Schapira DV (1995). Timing of weight gain and breast cancer risk. Cancer.

[B23] Bernstein L, Ross RK (1993). Endogenous hormones and breast cancer risk. Epidemiol Rev.

[B24] Narod SA (2001). Hormonal prevention of hereditary breast cancer. Ann N Y Acad Sci.

[B25] Cullinane CA, Lubinski J, Neuhausen SL, Ghadirian P, Lynch HT, Isaacs C, Weber B, Moller P, Offit K, Kim-Sing C (2005). Effect of pregnancy as a risk factor for breast cancer in BRCA1/BRCA2 mutation carriers. Int J Cancer.

[B26] WHO Expert Committee (1995). Physical Status The Use and Interpretation of Anthropometry.

[B27] Forbes GB, Reina JC (1970). Adult lean body mass declines with age: some longitudinal observations. Metabolism.

[B28] Ziegler RG (1997). Anthropometry and breast cancer. J Nutr.

[B29] Huang Z, Hankinson SE, Colditz GA, Stampfer MJ, Hunter DJ, Manson JE, Hennekens CH, Rosner B, Speizer FE, Willett WC (1997). Dual effects of weight and weight gain on breast cancer risk. JAMA.

[B30] Trentham-Dietz A, Newcomb PA, Egan KM, Titmus-Ernstoff L, Baron JA, Storer BE, Stampfer M, Willett WC (2000). Weight change and risk of postmenopausal breast cancer (United States). Cancer Causes Control.

[B31] Feigelson HS, Jonas CR, Teras LR, Thun MJ, Calle EE (2004). Weight gain, body mass index, hormone replacement therapy, and postmenopausal breast cancer in a large prospective study. Cancer Epidemiol Biomarkers Prev.

[B32] Cold S, Hansen S, Overvad K, Rose C (1998). A woman's build and the risk of breast cancer. Eur J Cancer.

[B33] Frank TS, Deffenbaugh AM, Reid JE, Hulick M, Ward BE, Lingenfelter B, Gumpper KL, Scholl T, Tavtigian SV, Pruss DR (2002). Clinical characteristics of individuals with germline mutations in BRCA1 and BRCA2: analysis of 10,000 individuals. J Clin Oncol.

[B34] Hilakivi-Clarke LA, Luoto R, Kinnunen T, Huttunen T, Gissler M, Koskenvuo M, Hemminki E (2002). Pregnancy weight gain and mother's breast cancer risk. 93rd Annual Meeting of the American Association for Cancer Research, San Francisco, CA.

[B35] Mueller WH (1982). The changes with age of the anatomical distribution of fat. Soc Sci Med.

[B36] Stoll BA (1995). Timing of weight gain in relation to breast cancer risk. Ann Oncol.

[B37] Schapira DV, Kumar NB, Lyman GH, Cox CE (1990). Abdominal obesity and breast cancer risk. Ann Intern Med.

[B38] Chang-Claude J, Becher H, Eby N, Gastert G, Wahrendorf J, Hamann U (1997). Modifying effect of reproductive risk factors on the age at onset of breast cancer for German BRCA1 mutation carriers. J Cancer Res Clin Oncol.

[B39] Kirschner MA, Ertel N, Schneider G (1981). Obesity, hormones, and cancer. Cancer Res.

[B40] Carroll KK (1998). Obesity as a risk factor for certain types of cancer. Lipids.

[B41] Potischman N, Swanson CA, Siiteri P, Hoover RN (1996). Reversal of relation between body mass and endogenous estrogen concentrations with menopausal status. J Natl Cancer Inst.

[B42] Henderson BE, Ross RK, Judd HL, Krailo MD, Pike MC (1985). Do regular ovulatory cycles increase breast cancer risk?. Cancer.

[B43] Pike MC, Krailo MD, Henderson BE, Casagrande JT, Hoel DG (1983). 'Hormonal' risk factors, 'breast tissue age' and the age-incidence of breast cancer. Nature.

[B44] MacDonald PC, Edman CD, Hemsell DL, Porter JC, Siiteri PK (1978). Effect of obesity on conversion of plasma androstenedione to estrone in postmenopausal women with and without endometrial cancer. Am J Obstet Gynecol.

[B45] Bruning PF, Bonfrer JM, Hart AA (1985). Non-protein bound oestradiol, sex hormone binding globulin, breast cancer and breast cancer risk. Br J Cancer.

[B46] Kaaks R (1996). Nutrition, hormones, and breast cancer: is insulin the missing link?. Cancer Causes Control.

[B47] Stoll BA (1999). Perimenopausal weight gain and progression of breast cancer precursors. Cancer Detect Prev.

[B48] Del Giudice ME, Fantus IG, Ezzat S, McKeown-Eyssen G, Page D, Goodwin PJ (1998). Insulin and related factors in premenopausal breast cancer risk. Breast Cancer Res Treat.

[B49] Campagnoli C, Biglia N, Belforte P, Botta D, Pedrini E, Sismondi P (1992). Post-menopausal breast cancer risk: oral estrogen treatment and abdominal obesity induce opposite changes in possibly important biological variables. Eur J Gynaecol Oncol.

[B50] Okosun IS, Liao Y, Rotimi CN, Prewitt TE, Cooper RS (2000). Abdominal adiposity and clustering of multiple metabolic syndrome in White, Black and Hispanic Americans. Ann Epidemiol.

[B51] Evans DJ, Hoffmann RG, Kalkhoff RK, Kissebah AH (1983). Relationship of androgenic activity to body fat topography, fat cell morphology, and metabolic aberrations in premenopausal women. J Clin Endocrinol Metab.

[B52] Kaye SA, Folsom AR, Soler JT, Prineas RJ, Potter JD (1991). Associations of body mass and fat distribution with sex hormone concentrations in postmenopausal women. Int J Epidemiol.

[B53] Rose DP, Gilhooly EM, Nixon DW (2002). Adverse effects of obesity on breast cancer prognosis, and the biological actions of leptin (review). Int J Oncol.

[B54] Hu X, Juneja SC, Maihle NJ, Cleary MP (2002). Leptin – a growth factor in normal and malignant breast cells and for normal mammary gland development. J Natl Cancer Inst.

[B55] Verkasalo PK, Thomas HV, Appleby PN, Davey GK, Key TJ (2001). Circulating levels of sex hormones and their relation to risk factors for breast cancer: a cross-sectional study in 1092 pre- and postmenopausal women (United Kingdom). Cancer Causes Control.

[B56] Moore JW, Key TJ, Bulbrook RD, Clark GM, Allen DS, Wang DY, Pike MC (1987). Sex hormone binding globulin and risk factors for breast cancer in a population of normal women who had never used exogenous sex hormones. Br J Cancer.

[B57] Renehan AG, Zwahlen M, Minder C, O'Dwyer ST, Shalet SM, Egger M (2004). Insulin-like growth factor (IGF)-I, IGF binding protein-3, and cancer risk: systematic review and meta-regression analysis. Lancet.

[B58] Werner H, LeRoith D (1996). The role of the insulin-like growth factor system in human cancer. Adv Cancer Res.

[B59] Khandwala HM, McCutcheon IE, Flyvbjerg A, Friend KE (2000). The effects of insulin-like growth factors on tumorigenesis and neoplastic growth. Endocr Rev.

[B60] Yu H, Rohan T (2000). Role of the insulin-like growth factor family in cancer development and progression. J Natl Cancer Inst.

[B61] Klauwer D, Blum WF, Hanitsch S, Rascher W, Lee PD, Kiess W (1997). IGF-I, IGF-II, free IGF-I and IGFBP-1, -2 and -3 levels in venous cord blood: relationship to birthweight, length and gestational age in healthy newborns. Acta Paediatr.

[B62] Ogilvy-Stuart AL, Hands SJ, Adcock CJ, Holly JM, Matthews DR, Mohamed-Ali V, Yudkin JS, Wilkinson AR, Dunger DB (1998). Insulin, insulin-like growth factor I (IGF-I), IGF-binding protein-1, growth hormone, and feeding in the newborn. J Clin Endocrinol Metab.

[B63] Juul A, Bang P, Hertel NT, Main K, Dalgaard P, Jorgensen K, Muller J, Hall K, Skakkebaek NE (1994). Serum insulin-like growth factor-I in 1030 healthy children, adolescents, and adults: relation to age, sex, stage of puberty, testicular size, and body mass index. J Clin Endocrinol Metab.

[B64] Okasha M, McCarron P, Gunnell D, Smith GD (2003). Exposures in childhood, adolescence and early adulthood and breast cancer risk: a systematic review of the literature. Breast Cancer Res Treat.

[B65] Collaborative Group on Hormonal Factors in Breast Cancer (2001). Familial breast cancer: collaborative reanalysis of individual data from 52 epidemiological studies including 58,209 women with breast cancer and 101,986 women without the disease. Lancet.

[B66] Stunkard AJ, Albaum JM (1981). The accuracy of self-reported weights. Am J Clin Nutr.

[B67] Weaver TW, Kushi LH, McGovern PG, Potter JD, Rich SS, King RA, Whitbeck J, Greenstein J, Steller TA (1996). Validation study of self-reported measures of fat distribution. Int J Obes Relat Metab Disord.

[B68] Rimm EB, Stampfer MJ, Colditz GA, Chute CG, Litin LB, Willett WC (1990). Validity of self-reported waist and hip circumferences in men and women. Epidemiology.

[B69] Troy LM, Hunter DJ, Manson JE, Colditz GA, Stampfer MJ, Willett WC (1995). The validity of recalled weight among younger women. Int J Obes Relat Metab Disord.

[B70] Must A, Willett WC, Dietz WH (1993). Remote recall of childhood height, weight, and body build by elderly subjects. Am J Epidemiol.

[B71] Stevens J, Keil JE, Waid LR, Gazes PC (1990). Accuracy of current, 4-year, and 28-year self-reported body weight in an elderly population. Am J Epidemiol.

[B72] Casey VA, Dwyer JT, Coleman KA, Krall EA, Gardner J, Valadian I (1991). Accuracy of recall by middle-aged participants in a longitudinal study of their body size and indices of maturation earlier in life. Ann Hum Biol.

